# Metabonomic Evaluation of Chronic Unpredictable Mild Stress-Induced Changes in Rats by Intervention of Fluoxetine by HILIC-UHPLC/MS

**DOI:** 10.1371/journal.pone.0129146

**Published:** 2015-06-16

**Authors:** Longshan Zhao, Zhili Xiong, Xiumei Lu, Shuning Zheng, Fang Wang, Lin Ge, Guangyue Su, Jingyu Yang, Chunfu Wu

**Affiliations:** 1 School of Pharmacy, Shenyang Pharmaceutical University, Shenyang, Liaoning, China; 2 Department of Pharmacology, Shenyang Pharmaceutical University, Shenyang, Liaoning China; 3 Department of Traditional Chinese Materia Medica, Shenyang Pharmaceutical University, Shenyang, China; Imperial College London, UNITED KINGDOM

## Abstract

Hydrophilic interaction-ultra high performance liquid chromatography (HILIC-UHPLC) allows the analysis of highly polar metabolites, providing complementary information to reversed-phase (RP) chromatography. By optimization of the preparation and analytical conditions in HILIC mode, HILIC-UHPLC/MS was applied for the global metabolic profiling of rat plasma samples generated in an experimental model of chronic unpredictable mild stress (CUMS), and the concomitant investigation of the protective effect of fluoxetine was also evaluated. Identification of plasma metabolic profiles indicated that significant changes in specific metabolites occurred after fluoxetine exposure, including increased phenylalanine, serine, acetyl-L-carnitine, carnitine and decreased creatine, betaine, proline, tryptophan, tyrosine, C16:0 LPC. Some novel biomarkers from this HILIC-UHPLC/MS approach were betaine, proline, tyrosine creatine and serine compared with the results of RP-UHPLC/MS. The complementary nature of this technique is confirmed and is on agreement with previously published studies.

## Introduction

Metabonomics is a top-down systems biology approach in which metabolic responses to biological interventions or disease process are analyzed and modeled **[1, 2].** During the past few years, metabonomics has been widely used in disease diagnosis, toxicological mechanism and the identification of biomarkers **[3,4].** Nowadays, global metabolic profiling can be typically performed using nuclear magnetic resonance (NMR) **[5,6]**, gas chromatograph/mass spectrometry(GC-MS) **[7,8]** and liquid chromatograph/mass spectrometry (LC-MS) **[9,10]**. Most recently, the application of UHPLC/MS has been expanded rapidly, which showed enormous potential for metabonomic study. The majority of metabolic profiling applications are based on reversed-phase (RP) chromatographic methods, however, for many endogenous metabolites, such as amino acids, nucleocide, nucleic acids and organic acids, which are expected to be high polar analytes and often poorly retained on RP columns **[11]**.

Hydrophilic interaction chromatography (HILIC) was firstly introduced by Alpert et al **[12]** to allow for the separation of polar moleculars. The combination of HILIC and MS detector can significantly expand the number of detected analytes with more comprehensive metabolite coverage. Therefore, HILIC-UHPLC/MS has been applied lately extensively into metabonomics as a highly complementary tool for metabolic profiling approach in recent years [13–15]. Evans et al [16] applied RP- and HILIC-technology combined with MS platform for untargeted metabolomics of acute respiratory distess syndrome, only one compound named l-lactate from the 44 total identified compounds was identified by both of the two technologies, which indicated that the technology of HILIC/MS was a complementary analytical platform for conventional RPLC/MS that can detect a broad range of metabolites.

The metabonomics study of CUMS model of depression has already been studied on the same sample by ^1^HNMR and UHPLC-RP/MS **[17]**. Here, we developed a HILIC-UHPLC/MS method for the global polar metabolic profiling of plasma, so as to provide the supplement of the metabonomics study in depression study of CUMS model, which has been widely used for studying clinical depression and evaluating antidepressant effects of diverse drugs.

## Materials and Methods

### Materials and reagents

The reference standards of uric acid, deoxycytidine, 3-indolepropionic acid and lysophosphatidylcholines (C16:0 LPC, C18:0 LPC, C18:1 LPC) were supplied by Sigma Corporation (St. Louis, MO, USA). Phenylalanine, tryptophan, tyrosine, proline and serine were supplied by Tianjin Bodi Chemical Co., Ltd. (Tianjin, China). Creatine, betaine, taurine, carnitine, creatinine, hippuric acid, citrate and α-ketoglutaric acid were supplied by Sinopharm Chemcial Reagent Co. Ltd (Shanghai, China). Cholic acid was purchased from the National Institutes for Food and Drug Control (Beijing, China). Fluoxetine was provided by Lilly S.A. (Spain, France). Acetonitrile and formic acid of HPLC grade were purchased from Dikma Corporation (Richmond Hill, NY, USA). Water was purified by redistillation and filtered through 0.22 μm membrane filter before use. NaCsI was obtained from Sigma-Aldrich (MO, USA). All other chemicals were of analytical grade.

### Animals

Twenty-four male Sprague–Dawley (SD) rats (180–220 g) were purchased from the Laboratory Animal Center at Shenyang Pharmaceutical University, which were fed with certified standard diet and tap water *ad libitum*. Temperature and humidity were regulated at 21–23°C and 40–60%, respectively. A 12 h light/dark cycle was established. The study was carried out in strict accordance with the recommendations in the Guide for the Care and Use of Laboratory Animals of the State Committee of Science and Technology of People’s Republic of China. The protocol was approved by the Committee on the Ethics of Animal Experiments of the Shenyang Pharmaceutical Universtiy (Permit Number: SYXK: 2014–0004). The chronic unpredictable mild stress (CUMS) procedure was conducted as previously described **[17]**. Briefly, the animals were individually housed and repeatedly exposed to a set of CUMS stressor as follows: overnight illumination; food and water deprivation; food deprivation; damp bedding; water deprivation followed by exposure to an empty bottle for 1 h; cage tilt; white noise of 85 dB; paired housing; and behavior restriction. Sucrose consumption was assessed once a week throughout the whole experiment period, which was used as an important indicator when evaluating whether the CUMS model is established successfully. After one week of acclimatization, the rats were randomly divided into three groups (each consists of 8 rats): (A) control group, (B) chronic unpredictable mild stress group, (C) 10 mg/kg/d group of fluoxetine lasting for 2 weeks. The plasma samples were collected after 2 weeks administration and stored at -70°C until analysis.

### HILIC-UHPLC/MS analysis

An ACQUITY Ultra Performance Liquid Chromatography system coupled with a triple quadrupole mass spectrometer (Waters Corp., Milford, MA, USA) was used to achieve the separation of the analytes, which was performed on an ACQUITY UPLC BEH HILIC (50 mm × 2.1 mm, i.d., 1.7 μm, Waters Corp., Milford, MA, USA) coupled with a C_18_ guard column (4.0 mm×3.0 mm, 5 μm, Phenomenex, Torrance, CA, USA). The data acquisition and sample quantification were operated using MassLynx NT 4.1 software with QuanLynx program (Waters Corp., Milford, MA, USA). The mobile phase consisted of solvent A (0.2% formic acid in water) and solvent B (acetonitrile). The gradient elution program started at 5% A for 2 min, changed to 20% A over 2–10 min, changed to 50% A in the next 2.5 min, then returned to the initial condition and re-equilibrated for 3 min. The flow rate of mobile phase was set at 0.25 mL/min. The injection volume was 10 μL. The temperature of column and autosampler were maintained at 40°C and 4°C, respectively.

Mass spectra were acquired using electrospray ionization in positive ion mode and full scan mode. The mass spectrometric conditions were optimized as follows: Source temperature, 110°C; Capillary voltage, 3.0 kV; Cone voltage, 25 V; Desolvation temperature, 350°C; Cone gas flow 30 L/h. The mass spectrometer was operated in positive ion mode. MS data were acquired using full scan mode from *m/z* 100–1000. In MS/MS experiments, argon was employed as the collision gas and collision energy was set between 5 and 35 eV. NaCsI was used for mass correction. Data was collected in centroid mode.

### Sample preparation

Prior to analysis, the plasma samples were thawed at room temperature. Acetonitrile (100 μL) was added to plasma (50 μL) and vortex-mixed for 30 s, then centrifuged at 13 000 rpm for 10 min, supernatant was transferred to an autosampler vial kept at 4°C and an aliquot of 10 μL was injected for UHPLC/MS analysis.

### Data analysis

The data files were processed using the Micromass Markerlynx Applications Manager version 4.1 (Waters Corp., Milford, USA) for peak detection and alignment. All data including detection of the mass, retention time and intensity of the peaks eluted in each chromatogram were normalized to the sum total ion intensity per chromatogram to obtain the relative intensities of metabolites. The resulting 3-dimensional data, peak number (retention time and *m/z* pairs), sample name and normalized ion intensity were introduced to SIMCA-P software package (ver 10.0, Umetrics, Umea, Sweden) for principal component analysis (PCA) and partial least squares discriminant analysis (PLS-DA). Statistical analysis was performed by SPSS (version 16.0, Chicago, USA). Differences between groups were analyzed by one-way analysis of variance (one-way ANOVA) and Student’s *t*-test. P-values less than 0.05 were considered significant and values less than 0.01 were considered highly significant.

### Method validation

The methods were validated including the precision, repeatability, post-preparative stability and stability [18,19]. 50 μL of plasma sample were mixed to generate a pooled quality control (QC) sample. The precision of injection was examined by six replicated injections of the same QC sample, while the method repeatability by analyzing six different QC samples which were separately prepared. The post-preparative stability of sample was tested by analyzing six QC samples at autosampler (maintained at 4°C) for 24 h compared with fresh-prepared QC samples (n = 6) continuously in a single batch. The sample stability of the freeze (-80°C)-thaw (room temperature) process was evaluated by analyzing the QC samples undergone 1 to 3 freeze-thaw cycles together with the freshly thawed QC samples (*n* = 6) in a single batch. The system stability investigation was carried out by analyzing a QC sample every 10 experimental samples during the sample analysis. Five ions (min_*m/z*) of 6.0_227.0, 8.8_496.6, 7.3_115.9, 2.6_103.7 and 7.6_257.0 were selected to evaluate the relative standard deviation (RSD) of retention time, *m/z* and peak area.

### Identification of the endogenous metabolites

With regard to the choice of biomarkers, variables were selected based on a threshold of variable importance in the projection (VIP) value (VIP > 1.0) and S-plot from the PLS model. Low molecular-weight metabolites were analyzed and profiled from full scan mass spectra. And metabolites were identified by comparing their chromatographic retention time and MS/MS fragmentation characteristics with the available authentic references. Furthermore, full scan mass spectra of these metabolites addressing their characteristic masses were interpreted using available biochemical databases, such as KEGG (http://www.kegg.com/); METLIN (http://metlin-scripps.edu/).

## Results and Discussion

### Method validation

For method validation study, 50 μL plasma sample was pooled to generate a pooled quality control (QC) sample. The QC samples into the scores plot labeled by run order can be found in S1 Fig. The results are shown in Table 1, represented by the five ions in positive ion mode, which indicated that the method had good repeatability and stability.

**Table 1:** Instrumental precision, method repeatability and stabilities of system and plasma samples of HILIC-UHPLC/MS.

t_R_ *_m/z*	Precision (RSD, %)			Repeatability (RSD, %)			System stability (RSD, %)			Sample stability (RSD, %)		
	t_R_	*m/z*	area	t_R_	*m/z*	area	t_R_	*m/z*	area	A	B	C
6.0_227.0	0.64	0.026	4.0	1.3	0.028	5.2	2.0	0.035	7.6	4.9	-3.4	-7.4
8.8_496.6	0.35	0.017	6.3	0.69	0.020	8.9	1.2	0.024	13	6.4	9.6	12
7.3_115.9	0.23	0.016	2.7	0.38	0.018	4.5	0.66	0.020	6.0	5.8	4.3	6.8
2.6_103.7	0.27	0.024	8.9	0.44	0.026	9.4	0.89	0.026	13	-7.2	-8.5	-14
7.6_257.0	0.19	0.012	4.8	0.25	0.015	5.8	0.42	0.014	8.7	-5.3	4.6	4.8

Note: A, area at room temperature for 4 h; B, area by post preparative of 24 h; C, area after three freeze-thaw cycles.

### Biochemical alterations in CUMS-induced depression model in rats

**Fig 1:**
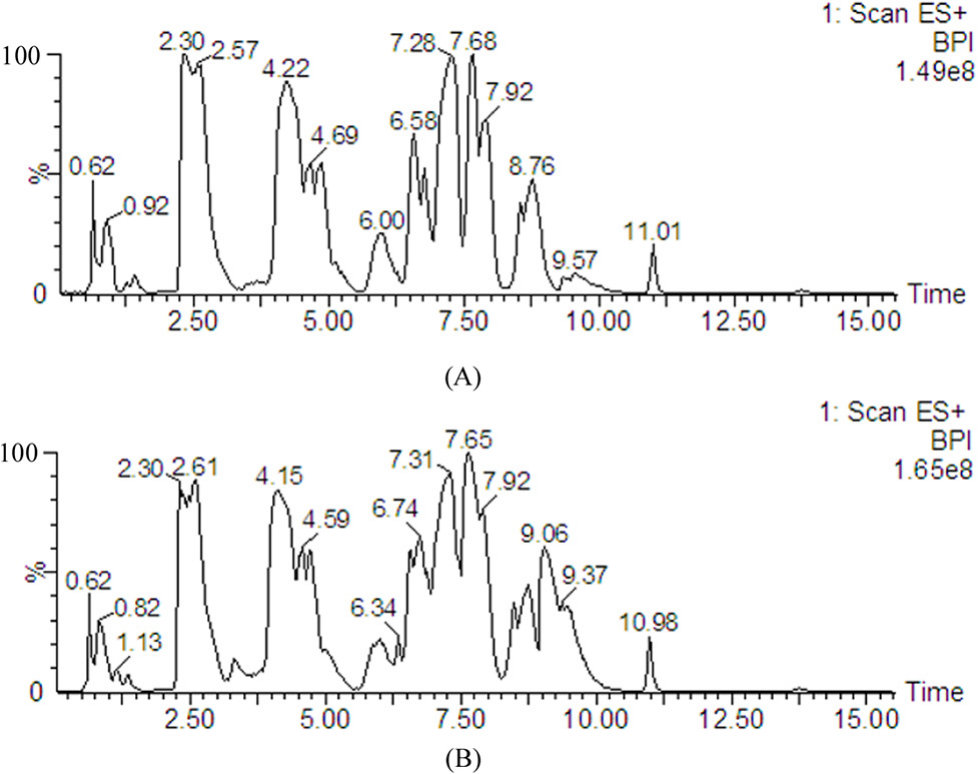
Base peak intensity (BPI) chromatograms of plasma metabolite profiles from (A) a control rat and (B) a model rat measured by HILIC-UHPLC/MS.

**Fig 2:**
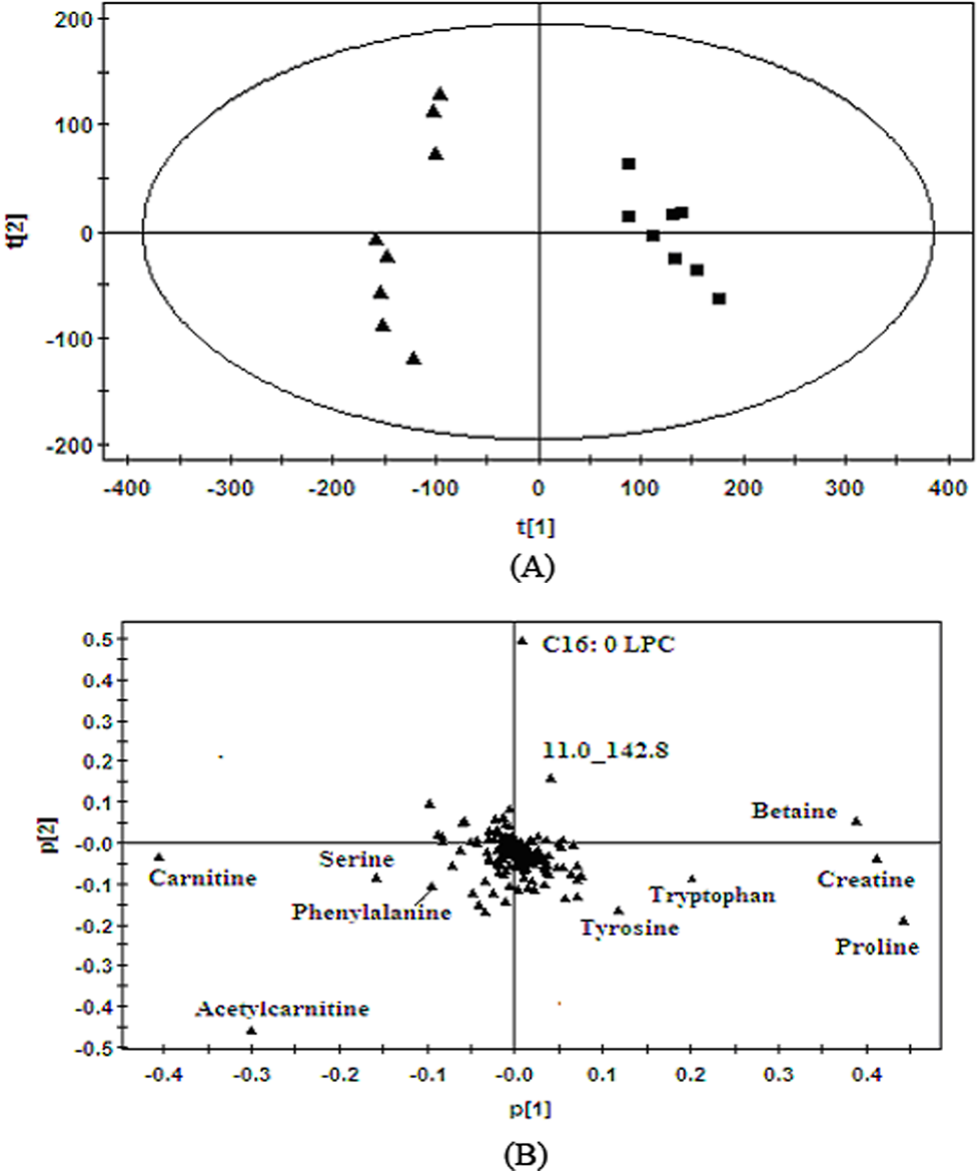
(A) Score plot and (B) loading plot derived from PCA model classifying control rats (■) and model rats (▲) based on plasma metabolite profiles measured by HILIC-UHPLC/MS.

Typical base peak intensity (BPI) chromatograms from a control rat and a CUMS model rat plasma by HILIC-UHPLC/MS in positive ion modes are shown in Fig 1. Based on the analysis of plasma metabolic data, a good separation between control rats and CUMS rats was achieved in PCA score plots according to the first component, which indicated that the significant metabolic changes were induced by CUMS vs control model. The first two principal components explained 75% of the total variances in positive ion modes (Fig 2A).

It could be concluded from the results of corresponding loading plots and ANOVA test (Fig 2B, Table 1), the concentrations of phenylalanine, serine, acetyl-L-carnitine and carnitine increased significantly in model rats compared with the control group, while creatine, betaine, proline, tryptophan, tyrosine, C16: 0 LPC and metabolite 11.0_142.8 decreased significantly in the model rats. The biomarkers in plasma with related to the depression by HILIC-UHPLC/MS were achieved and listed in Table 2 including the retention time, mass-to-charge ratio, metabolite identification, ANOVA analysis and changing trend.

**Table 2:** Biomarkers related to pathological mechanism of depression based on plasma metabolite profiles measured by HILIC-UHPLC/MS.

t_R_ (min)	m/z (amu)	Metabolite identification	ANOVA analysis (*p* Value)	Change trend compared with control rats
8.0	131.9	Creatine^a^	6.04 E-04	↓***
7.6	117.8	Betaine^a^	1.94 E-04	↓***
4.8	166.0	Phenylalanine^a^	3.09 E-02	↑*
7.3	115.9	Proline^a^	6.60 E-05	↓***
7.3	105.7	Serine^a^	1.46 E-04	↑***
4.4	204.9	Tryptophan^a^	1.10 E-04	↓***
6.6	181.9	Tyrosine^a^	4.31 E-04	↓***
7.6	203.9	Acetylcarnitine^b^	1.30 E-05	↑***
6.6	162.0	Carnitine^a^	3.88 E-05	↑***
8.8	496.6	C16: 0 LPC^a^	1.11 E-02	↓*
11.0	142.8	Unidentified	4.52 E-02	↓*

^a^Metabolites identified by comparing with database and authentic standards

^b^Metabolites identified by comparing with literatures and database resources

*p < 0.05

**p < 0.01 and

***p < 0.001.

### Intervention effects of fluoxetine

**Fig 3:**
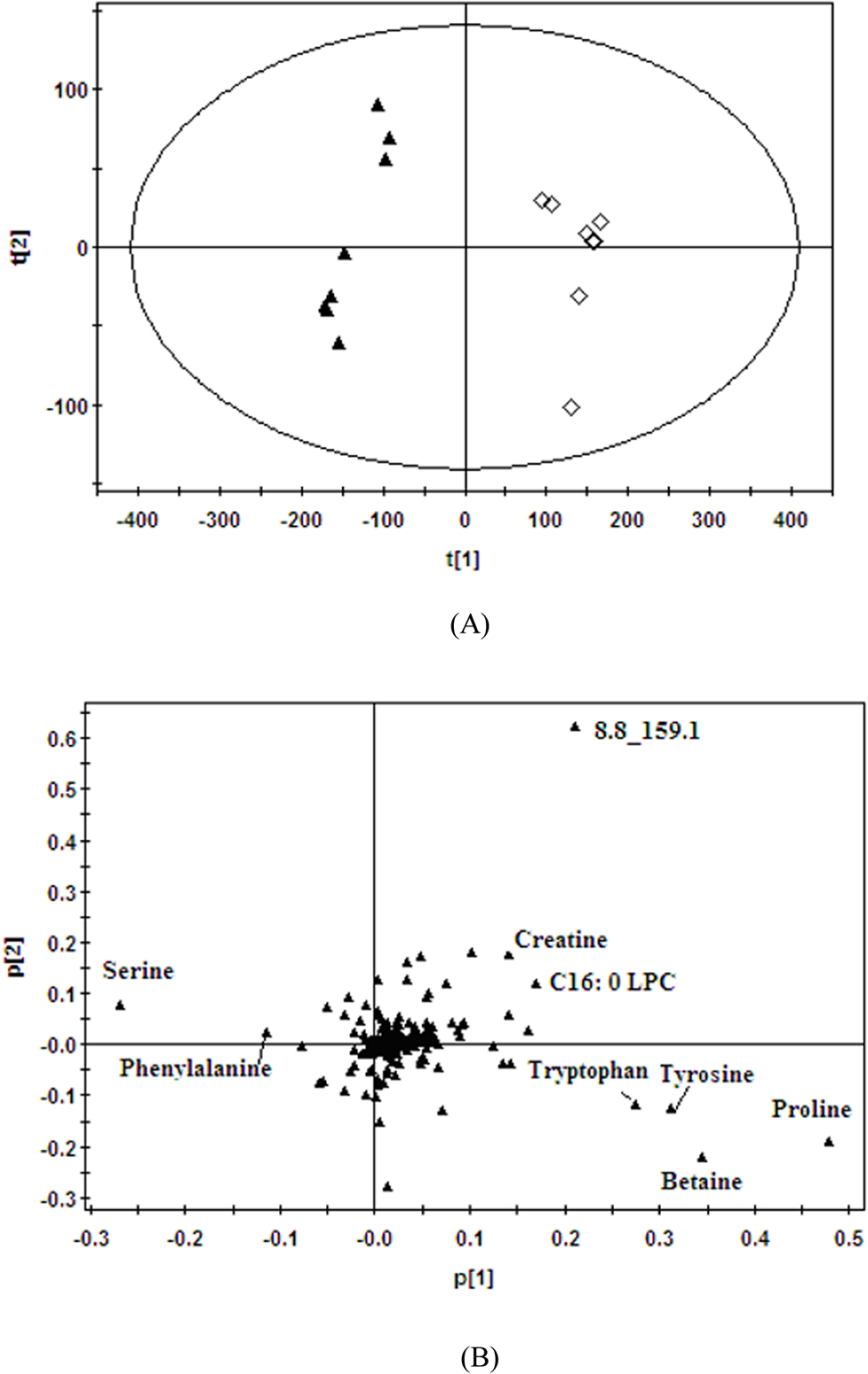
(A) Score plot and (B) loading plot derived from PCA model classifying (▲) model rats and (◇) fluoxetine treated rats based on plasma metabolite profiles measured by HILIC-UHPLC/MS.

PCA and PLS-DA are the two most popular pattern recognition methods to gain information for classification and identification of metabolites. PCA, an unsupervised method, can be applied as the first step in the separation procedure to filter out the noise and reduce the dimension of data to whiten the observation. While PLS-DA tends to improve the separation between groups compared to PCA, there is some risk that increased apparent separation can be an artifact of the PLS-DA algorithm and not reflect variances that truly distinguish between the groups [20]. In order to investigate the intervention effects of fluoxetine on the depression rats after simultaneous administration for two weeks, the plasma metabolic data from the model and fluoxetine group by HILIC-UHPLC/MS method were analyzed by PCA, which are depicted in **Fig 3**. It showed obvious separation between two groups, which indicates the plasma metabolites changed significantly after administration of fluoxetine for two weeks, the two components explained 79% of the total variances. Nine ions that contributed to the observed separation and showed significant difference in abundance between the two groups were found from loading plot **(Fig 3B)**, which were considered as the biomarkers related to the anti-depression actions. Their retention time, mass-to-charge ratio, metabolite identification, ANOVA analysis and changing trends are listed in **Table 3**. Among them eight biomarkers were identified as proline, betaine, tryptophan, tyrosine, creatine, serine, phenylalanine, C16:0 LPC.

**Table 3:** Biomarkers related to antidepressant mechanism of fluoxetine based on plasma metabolite profiles measured by HILIC-UHPLC/MS.

t_R_ (min)	m/z (amu)	Metabolite identification	ANOVA analysis (*p* Value)	Change trend compared with control rats
8.0	131.9	Creatine^a^	3.32 E-02	↑*
7.6	117.8	Betaine^a^	2.53 E-03	↑**
4.8	166.0	Phenylalanine^a^	4.36 E-03	↓**
7.3	115.9	Proline^a^	7.84 E-05	↑***
7.3	105.7	Serine^a^	6.25 E-05	↓***
4.4	204.9	Tryptophan^a^	1.18 E-03	↑**
6.6	181.9	Tyrosine^a^	4.22 E-04	↑***
8.8	496.6	C16: 0 LPC^a^	4.10 E-02	↑*
8.8	159.1	Unidentified	4.12 E-03	↑**

^a^Metabolites identified by comparing with database and authentic standards

^b^Metabolites identified by comparing with literatures and database resources

*p < 0.05

**p < 0.01 and

***p < 0.001.

**Fig 4:**
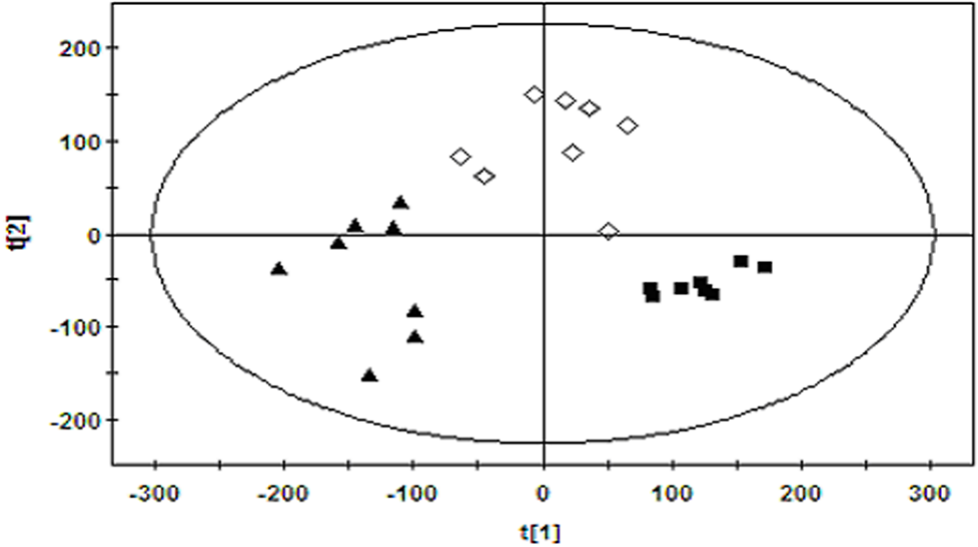
Score plot derived from PLS-DA model classifying (■) control rats, (▲) model rats and (◇) fluoxetine treated rats based on plasma metabolite profiles measured by HILIC-UHPLC/MS.

The PLS-DA analysis was derived from the data of group A, B and C with the following parameters: R^2^Y = 0.8, Q^2^ = 0.704, R^2^ intercept = 0.175, Q^2^ intercept = -0.3. From the Fig 4, fluoxetine group were between model group and control group according to the first component, which suggested that fluoxetine may help model group to return to the normal state.

### Biomarker elucidation

The results indicated that the plasma concentration of phenylalanine, serine, carnitine and acetyl-L-carnitine in group B increased significantly compared with that in group I, while creatine, betaine, proline, tryptophan, tyrosine and C16: 0 LPC decreased significantly, which were related to the pathomechanism of depression. Among them, tryptophan, and C16:0 LPC have been discussed previously [21].

The major metabolic pathway of phenylalanine is metabolited into tyrosine by the action of phenylalanine hydroxylase. The concentration of phenylalanine in CUMS model group increased significantly, while the concentration of tyrosine decreased significantly, and the ratio of phenylalanine and tyrosine increased, indicated that the bioactivity of phenylalanine hydroxylase decreased, which was consistent with the report [22]. The essential accessory factor for phenylalanine hydroxylase is tetrahydrobiopterin (BH4), while it was reported that the BH4 decreased in the serum of depression patients [23]. Therefore, the absence of BH4 might result in the decrease of activity for phenylalanine hydroxylase, then to prevent phenylalanine from converting to tyrosine, so as to increase phenylalanine and decrease tyrosine. The tyrosine might be metabolited into dihydroxyphenylanaline by tyrosine hydroxylase, and then further metabolited into dopamine (DA), norepinephrine (NE) and epinephrine (E), which were the precursor of catecholamine neurotransmitters. The decrease of tyrosine in model group can lead to the decrease of catecholamine neurotransmitters, which are consist with the catecholamine hypothesis of depression [24].

The results indicated that serine in group B increased. It was reported that serine in plasma was mainly originated in the conversion of synthesis of glycine in kidney, the glycine was transformed into serine by serine hydroxymethyl transferase, affective disorders was closely related to the interconversion injury of glycine-serine **[25]**, and the high plasma concentration of serine has been the potential biomarker for various mental illness such as depression, schizophrenia and mania, etc [26]. The significant increase of serine might be due to the increase of glycine-serine interconversion, which was consistent with the results of the increased ratio of serine and glycine in depression patients **[27]**.

The results indicated that proline in group B was significant lower than that in group A. Proline can be metabolited into pyruvate, the latter was converted into the important enter molecule in the tricarboxylic acid (TCA) cycle namely acetyl coenzyme A, therefore proline can indirectly participate in the TCA cycle. The decreasing plasma concentration of proline will cause the decrease of pyruvate and weakness of TCA cycle, which was related to the inadequate energy supply.

The significant decrease of creatine in group B can make the disorder of energy metabolism, which are the important pathological mechanisms of depression. Creatine is widely distributed in the muscles, brain, heart and other tissues, and can be catalyzed by creatine kinase into phosphocreatine and adensine diphosphate (ADP), making the creatine-creatine phosphate system under dynamic equilibrium. The creatine-creatine phosphate system can regulate the energy homeostasis by the action of energy buffer, energy transport and metabolic regulation **[28, 29]**. Creatine can benefit the supplement of energy in brain, in order to maintain the ATP levels of nerve cell activity. In the present investigation, the significant decrease of plasma creatine can lead to the inadequate energy supply of brain, so as to influence the nerve cell activity, and finally cause the depression.

Plasma betaine concentrations in group B were significantly lower than that in group A. Betaine participate in synthesis of the homocysteine, after demethylation by cysteine methyltransferase which can transformed into methionine, therefore the availability of betaine might be the main dependent index for the concentration of homocysteine. The significant decrease of betaine in group B can inhibit the conversion reaction of homocysteine, then to increase the concentration of homocysteine. It was reported that homocysteine increased significantly in the plasma and serum of depression patients [30], what’s more, the high level of homocysteine was significant related to the severity of depression [31]. Homocysteine can participate in the oxidative stress, destroy the normal redox state of nerve cell, influence the redox signal path, which were the risk factors of depression with neurotoxicity [32]. The supplement of betaine can convert the homocysteine into methionine, decrease the plasma concentration of homocysteine, which has been used for the prevention and treatment of depression.

The main function of carnitine and acetyl carnitine is to carry and transfer the activated fatty acid into mitochondria by β-oxidation, so as to regulate the balance of fatty acid metabolism. The abnormal concentration of carnitine and acetyl carnitine in group B domonstrated the disorders of fatty acid metabolism in body.

Fluoxetine can significantly increase the plasma concentration of creatine, betaine, proline and C16: 0 LPC, significantly decrease the plasma concentration of phenylalanine. Otherwise, fluoxetine can significantly increase the concentration of tryptophan and tyrosine, decrease the plasma concentration of serine. The addition of creatine and proline in plasma can directly strengthen the creatine-creatine phosphate system, so as to indirectly strengthen TCA cycle, increase the energy supply and regulate the disorders of energy metabolism. It can also significantly increase the plasma concentration of betaine, promote the homocysteine convert to methionine, decrease the neurotoxicity of homocysteine. What’s more, it can also regulate various metabolic pathways to play antidepressant effects. Fluoxetine can regulate the abnormal amino acid metabolism, significantly decrease the phenylalanine and serine in plasma, increase tyrosine and decrease the transformation of serine by glycine, make more phenylalanine convert into tyrosine, so as to increase the synthesis of catecholamine neurotransmitters, and finally improve the depression.

### Comparison between HILIC- and RP-UHPLC/MS

**Fig 5:**
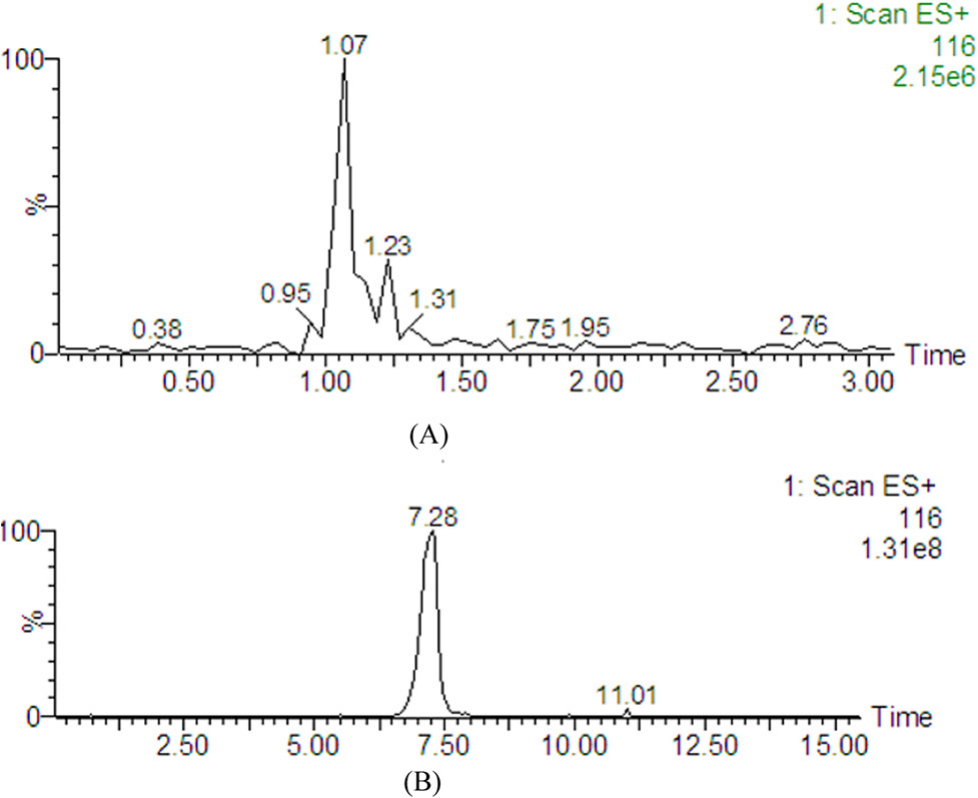
Extracted ion chromatograms of proline from plasma metabolite profiles measured by (a) RP-UPLC-MS and (b) HILIC-UPLC-MS.

The conventional RP-UHPLC/MS can only retain and separate metabolites with weak and middle polarity, however, the application of high organic content mobile phase has made HILIC-UHPLC/MS an attractive complementary tool to the widely used reversed-phase chromatographic separations in metabonomic studies. Fig 5 illustrated that the retention time of proline on RP-UHPLC column is 1.1 min with poor peak shape and weak chromatographic retention, and significant influence of the endogenous substances, while it was eluted at 7.3 min with better peak shape and resolution for HILIC-UHPLC mode (chromatographic conditions can be found in S1 Table). What’s more, the higher concentration of organic solvent in mobile phase can improve the ionization efficiency of ESI source, achieving better mass response. Which can be seen in Fig 5, the mass response was significantly increased from 2.15e^6^ for RP-UHPLC/MS to 1.31e^8^ for HILIC-UHPLC/MS. For RP-UHPLC/MS, a wealth of information can be provided by both positive and negative modes, therefore both modes were applied in the metabonomics study (S2 Fig), while for HILIC-UHPLC/MS, more metabolite information obtained in positive mode than that in negative mode, then only positive mode was finally applied.

Biomarkers related to antidepressant mechanism by RP-UHPLC/MS were the metabolites with weak polarity, such as cholic acid, LPC etc (S2 and S3 Tables), while other biomarkers with strong polarity, such as serine, proline, tyrosine and carnitine, can be concluded by RP-UHPLC/MS. The results indicated that the HILIC mode can provide as a supplement to the conventional RP-UHPLC/MS metabonomics study.

## Conclusions

A metabonomics method based on HILIC-UHPLC/MS has been developed to study the influence of fluoxetine on CUMS rats. A clear separation was observed among control group, CUMS group and CUMS treated by fluoxetine group. Some potential biomarkers like phenylalanine, serine, acetyl-L-carnitine, carnitine, creatine, betaine, proline, tryptophan, tyrosine, C16:0 LPC have been identified. Compared with conventional RPLC method, some novel biomarkers like betaine, proline, tyrosine creatine and serine were confirmed by HILIC-UHPLC/MS approach. This work shows that HILIC-UHPLC/MS is a valuable tool for drug metabolism.

## Supporting Information

S1 Fig (A) Score plots derived from PCA model classifying QC sample(△), control rats (■) and model rats (▲). (TIF)

S2 Fig (A) Positive and (B) negative ion base peak intensity (BPI) chromatograms of plasma metabolite profiles from a control rat measured by RP-UPLC-MS. (TIF)

S1 Table Gradient elution program for plasma metabolite profile based on RP-UPLC-MS. (DOC)

S2 Table Biomarkers related to pathological mechanism of depression based on plasma metabolite profiles measured by RP-UPLC-MS. (DOC)

S3 Table Biomarkers related to antidepressant mechanism of fluoxetine based on plasma metabolite profiles measured by RP-UPLC-MS. (DOC)

S1 Dataset Selected original metabonomics data. (ZIP)
