# Supplementary figures and images for: Metabonomic Evaluation of Chronic Unpredictable Mild Stress-Induced Changes in Rats by Intervention of Fluoxetine by HILIC-UHPLC/MS
Source: PLoS One. 2015 Jun 16;10(6):e0129146. doi: 10.1371/journal.pone.0129146 (PMC4469692; doi:10.1371/journal.pone.0129146)

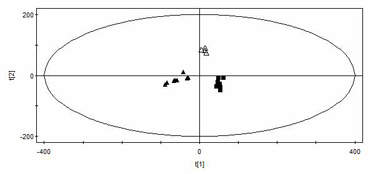

Supplement: S1 Fig — (TIF) [file pone.0129146.s001.tif]

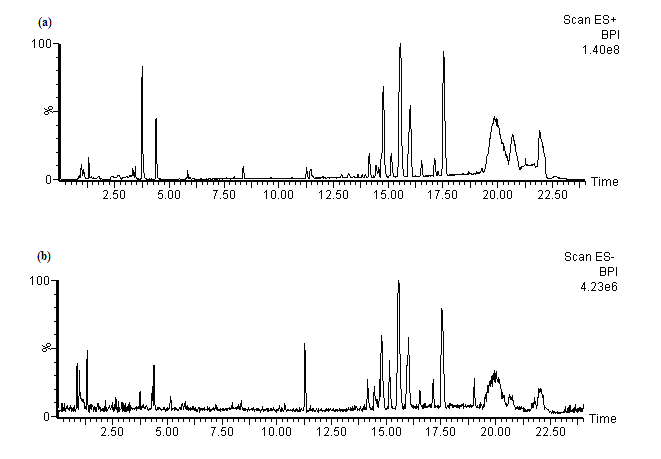

Supplement: S2 Fig — (TIF) [file pone.0129146.s002.tif]
