# Supplementary material for: Metabonomic Evaluation of Chronic Unpredictable Mild Stress-Induced Changes in Rats by Intervention of Fluoxetine by HILIC-UHPLC/MS
Source: PLoS One. 2015 Jun 16;10(6):e0129146. doi: 10.1371/journal.pone.0129146 (PMC4469692; doi:10.1371/journal.pone.0129146)
Supplement: S1 Table — (DOC) [file pone.0129146.s003.doc]

Table S1

Gradient elution program for plasma metabolite profile based on RP-UPLC-MS

| **Time (min)** | **A (%)** | **B (%)** | **Curve** |
| --- | --- | --- | --- |
| initial | 100 | 0 | - |
| 2 | 100 | 0 | 6 |
| 20 | 5 | 95 | 6 |
| 21 | 5 | 95 | 6 |
| 24 | 100 | 0 | 1 |

Conditions: ACQUITY UPLC BEH C_18_ (100 mm × 2.1 mm, i.d., 1.7 μm， Waters）A: 0.1% formic acid in water B: 0.1% formic acid in acetonitrile
