# Supplementary material for: Metabonomic Evaluation of Chronic Unpredictable Mild Stress-Induced Changes in Rats by Intervention of Fluoxetine by HILIC-UHPLC/MS
Source: PLoS One. 2015 Jun 16;10(6):e0129146. doi: 10.1371/journal.pone.0129146 (PMC4469692; doi:10.1371/journal.pone.0129146)
Supplement: S2 Table — (DOC) [file pone.0129146.s004.doc]

Table S2 Biomarkers related to pathological mechanism of depression based on plasma metabolite profiles measured by RP-UPLC-MS

| **Retention time (min)** | **m/z (amu)** | **Metabolite identification** | **ANOVA analysis**  **(*p* Value)** | **Change trend compared with control rats** |
| --- | --- | --- | --- | --- |
| **Positive ion mode** | | | | |
| 4.4 | 188.3 | Tryptophan fragment ^a^ | 1.28 E-05 | ↓^***^ |
| 8.4 | 130.0 | 3-IPA fragment ^a^ | 5.68 E-05 | ↓^***^ |
| 3.7 | 120.0 | Phenylalanine fragment ^a^ | 2.48 E-03 | ↑^**^ |
| 1.3 | 112.0 | Deoxycytidine ^a^ | 1.59 E-03 | ↑^**^ |
| 14.8 | 544.6 | C20: 4 LPC ^b^ | 2.61 E-04 | ↑^***^ |
| 13.6 | 468.6 | C14: 0 LPC ^b^ | 9.58 E-05 | ↓^***^ |
| 15.6 | 496.6 | C16: 0 LPC ^a^ | 1.97 E-03 | ↓^**^ |
| 11.6 | 274.2 | Unidentified | 1.38 E-02 | ↓^*^ |
| 13.1 | 415.1 | Unidentified | 4.06 E-02 | ↑^*^ |
| **Negative ion mode** | | | | |
| 4.4 | 203.2 | Tryptophan ^a^ | 5.70 E-05 | ↓^***^ |
| 1.4 | 167.1 | Uric acid ^a^ | 5.90 E-06 | ↓^***^ |
| 3.7 | 164.2 | Phenylalanine ^a^ | 4.96 E-03 | ↑^**^ |
| 11.3 | 407.5 | Cholic acid ^a^ | 1.74 E-02 | ↑^*^ |
| 14.6 | 612.6 | C22: 6 LPC ^b^ | 3.09 E-04 | ↑^***^ |
| 14.8 | 588.6 | C20: 4 LPC ^b^ | 6.73 E-04 | ↑^***^ |
| 15.6 | 540.6 | C16: 0 LPC ^a^ | 2.33 E-03 | ↓^**^ |
| 15.7 | 452.1 | Unidentified | 7.40 E-03 | ↓^**^ |
| 17.8 | 480.0 | Unidentified | 4.76 E-02 | ↓^*^ |

^a^Metabolites identified by comparing with database and authentic standards.

^b^Metabolites identified by comparing with literatures and database resources.

**p* < 0.05, ***p* < 0.01 and ****p* < 0.001.
